# Women Neuroscientist Disciples of Pío del Río-Hortega: the Cajal School Spreads in Europe and South America

**DOI:** 10.3389/fnana.2021.666938

**Published:** 2021-05-10

**Authors:** Cristina Nombela, Emilio Fernández-Egea, Elena Giné, Yulia Worbe, Juan del Río-Hortega Bereciartu, Fernando de Castro

**Affiliations:** ^1^Biological and Health Psychology Department, Universidad Autónoma de Madrid, Madrid, Spain; ^2^Department of Psychiatry, Behavioural and Clinical Neuroscience Institute, University of Cambridge, Cambridge, United Kingdom; ^3^Departamento de Biología Celular, Universidad Complutense de Madrid, Madrid, España; ^4^Department of Neurophysiology, Saint-Antoine Hospital, Sorbonne Université, Paris, France; ^5^Departamento de Pediatría, Inmunología, Obstetricia-Ginecología, Nutrición-Bromatología, Psiquiatría e Historia de la Medicina, Universidad de Valladolid, Valladolid, Spain; ^6^Instituto Cajal-CSIC, Spanish Research Council/Consejo Superior de Investigaciones Científicas-CSIC, Madrid, Spain

**Keywords:** female neuroscientists, microglia, history of neuroscience, Spanish Neurological School, Amanda Pellegrino de Iraldi, Dorothy Russell, oligodendrocyte, pioneer female scientists

## Abstract

Pio del Rio-Hortega was not only the discoverer of the microglia and oligodendroglia but also possibly the most prolific mentor of all Santiago Ramon y Cajal’s disciples (Nobel awardee in Physiology or Medicine 1906 and considered as the father of modern Neuroscience). Among Río-Hortega’s mentees, three exceptional women are frequently forgotten, chronologically: Pio’s niece Asunción Amo del Río who worked with Río-Hortega at Madrid, Paris, and Oxford; the distinguished British neuropathologist Dorothy Russell who also worked with *Don* Pío at Oxford; and Amanda Pellegrino de Iraldi, the last mentee in his career. Our present work analyzes the figures of these three women who were in contact and collaborated with *Don* Pío del Río-Hortega, describing the influences received and the impact on their careers and the History of Neuroscience. The present work completes the contribution of women neuroscientists who worked with Cajal and his main disciples of the Spanish Neurological School both in Spain (previous work) and in other countries (present work).

## Introduction

Santiago Ramón y Cajal (1852–1934) is broadly acknowledged as the founding father of modern Neuroscience (Shepherd, 1991). Although he conducted most of his crucial discoveries on the fine structure of the nervous system on his own, Cajal’s growing international recognition convinced Spanish authorities to construct him and equip a laboratory, as well as to hire stable collaborators after 1902. These gave rise to what we know as the Spanish Neurological School or, more colloquially, the Cajal School, maybe “*the most fruitful school in the History of Biomedicine*,” in the words of Charles Sherrington (Sherrington, 1935; for general overviews on the contributions of the Cajal School to modern Neuroscience, see de Castro, 1981, 2019; Andres-Barquin, 2002). There were no data regarding relevant contributions from female researchers. It was not up to 2019 that we rescued the names of some women that worked in the Cajal School between 1911 and 1945. In particular, Laura Forster and Manuela Serra worked with Santiago Ramón y Cajal, while Soledad Ruiz-Capillas and M^*a*^ Luisa Herreros collaborated with two of the most prominent direct disciples of Cajal, Gonzalo R. Lafora and Fernando de Castro, respectively (Giné et al., 2019; Nombela et al., 2020). Nevertheless, our previous works circumscribed to the women neuroscientists that physically worked in the laboratory of Cajal, in Madrid. Further inquiry led us to notice three more women that worked with Pío del Río-Hortega out of Spain, in different stages of his exile during the Spanish Civil War (1936–1939) and after.

Río-Hortega was probably the most successful of the direct disciples of Cajal. He discovered that the “third element of the nervous system” was truly composed of microglia plus oligodendroglia (del Río-Hortega, 1919a,b,c,d, 1921; Sierra et al., 2016) and conceived the first histogenetic classification of brain tumors, among other significant discoveries (del Río-Hortega, 1932; Ramon and Cajal Agüeras, 2016). Consequently, in the 1920s, he was one of the most relevant neuroscientists in the world and his figure attracted many outstanding disciples, as the American neuropathologist and neurosurgeon Wilder S. Penfield (Penfield, 1924, 1977; del Río-Hortega and Penfield, 1927; see also de Castro, 1981, 2019; del Río-Hortega Bereciartu, 2020). Pío del Río-Hortega was proposed by first time for the Nobel Prize in Physiology or Medicine 1929^1^ because of his *”work on the histology and histopathology of the nervous system, especially studies of glial tissue (microglia and oligodendroglia)”* (de Castro, 2019; del Río-Hortega Bereciartu, 2020). Escaping from the Spanish Civil War (1936–1939) first, and the 2nd World War (1939–1945) later, Río-Hortega continued researching out of Spain up to his death. During that period, three of his closest collaborators were women: Asunción Amo del Río, Dr. Dorothy Russell, and Dr. Amanda Pellegrino de Iraldi.

## Materials and Methods

For the present work, we have comprehensively reviewed the English- and Spanish-language literature pertinent to the history of Pío del Río-Hortega (1882–1945) and his associations with the main characters of our study: Asuncíon Amo del Río, Dorothy S. Russell, and Amanda Pellegrino de Iraldi. This includes a relevant amount of the original research papers written by these authors and others. We have also exhaustively examined different archives in search of significant documents (including photographs), such as the Legado Cajal (Instituto Cajal-CSIC, Madrid, Spain), the Archivo Pío del Río-Hortega (Valladolid, Spain), and the Archivo científico Fernando de Castro (Madrid, Spain). All of them are included by the United Nations Educational, Scientific and Cultural Organization (UNESCO) in the collective entry “Archives of Santiago Ramón y Cajal and the Spanish Neurohistological School” as part of the Human Heritage http://www.unesco.org/new/en/communication-and-information/me mory-of-the-world/register/full-list-of-registered-heritage/regist ered-heritage-page-1/archives-of-santiago-ramon-y-cajal-and-th e-spanish-neurohistological-school/. Finally, different researchers who knew in person Amanda Pellegrino de Iraldi have been contacted in Canada and Argentina (see Acknowledgments).

## Results

### Asunción Amo del Río: Niece and Technician of Río-Hortega

**FIGURE 1:**
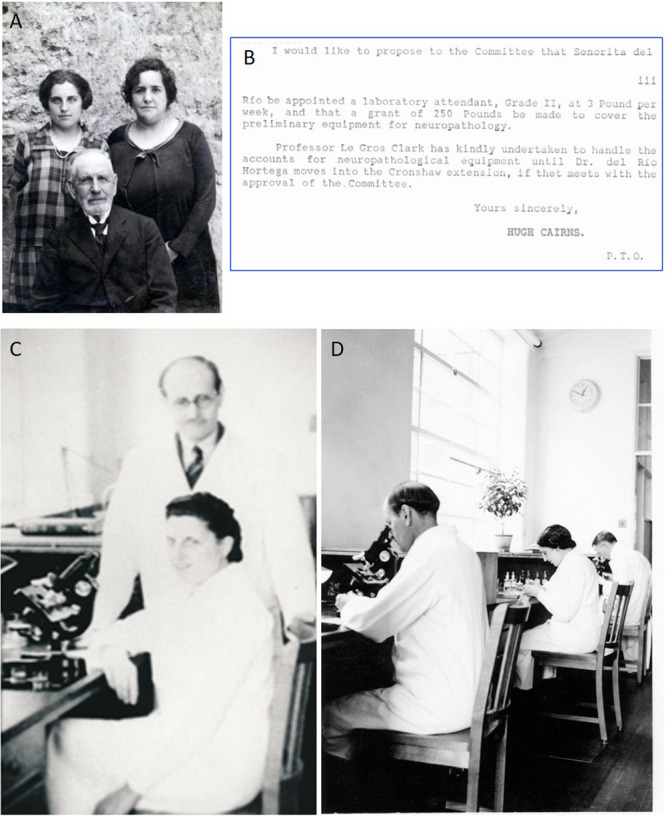
Asunción Amo del Río. **(A)** Young Asunción Amo del Río (on the left) with her mother and her grandfather Juan at Portillo (Valladolid). **(B)** Detail of the report by Hugh Cairns (see the signature, below) to the Nuffield Committee proposing the appointment of “Senorita [*sic*] Del Río” as laboratory attendant of Pío del Río-Hortega. **(C)** Río-Hortega (on feet) and his niece and attendant, Asunción Amo del Río, at the laboratory of Neuropathology of the Nuffield Department, University of Oxford. **(D)** Dr. Pío del Río-Hortega (on the left), Asunción Amo del Río (in the middle), and Dr. William Gibson (on the right) developing their work in Neuropathology at the Nuffield Department, in Oxford, 1938.

Asunción Amo del Río (1906–1995) was the oldest of the nieces of Río-Hortega (Figure 1A). Born in Portillo (Valladolid), she went to the school and high school in Valladolid and moved to Madrid in 1922 with other members of the family Río-Hortega (del Río-Hortega Bereciartu, 1993). Then a 16-year-old youngster, Asunción Amo started to work as an auxiliary technician under the direct orders of his uncle at the *Instituto Nacional del Cáncer* (National Institute for Cancer Research), founded in the early 1920s. Due to del Río-Hortega’s international reputation in the field of brain tumors, he took charge of a laboratory in 1928^2^. He combined it with the direction of the Laboratory of Neuropathology, founded by the *Junta para Ampliación de Estudios-JAE* at the *Residencia de Estudiantes* from 1920 (del Río-Hortega Bereciartu, 1993). Highly skilled in histological techniques, Asunción Amo was the main technical collaborator of Río-Hortega on the pathology of brain tumors (del Río-Hortega, 1933). She was entrusted to train the foreign visitors, like Karl Neuhaus (disciple of Aschoff), Lewis Stevenson, Bernard Alpers, Nathan Norcross, Lester King, William C. Gibson, and Alaistair Robb-Smith (the latter two were sent by Charles Sherrington and Hugh Cairns; Gibson, 1994).

In July 1936, a failed military *coup d’état* gave rise to the Spanish Civil War. The rebel nationalist troops were soon on the way to Spain’s capital, Madrid. In September, Pío del Río-Hortega attended an international meeting on cancer held in Brussels (Belgium) and stops at Paris on his way back: being there, he found to his amazement that he had been accused of the valuable radium stolen from the *Instituto Nacional del Cáncer*. Therefore, he came back to Madrid, made a recount of the amounts of radium, clarifying the false accusation, and closes down both laboratories (del Río-Hortega Bereciartu, 1993). Under the first bombs falling the *Instituto Nacional del Cáncer*, Asuncíón Amo helped her uncle and his close friend, Nicolás Gómez del Moral, to rescue some thousands of histological slides, microscopes, and other important research materials. Although all the three were unharmed, Asunción fell unconscious from the tension of the danger to which they had been exposed and she was evacuated in a military ambulance (de Viani and Jimenez Carmena, 1991; Iglesias-Rozas and Garrosa, 2013). This was just a few weeks before the building became a non-stop part of the front of war from November 1936, and by the end of the conflagration, on April 1, 1939, there was hardly any stone on stone. It was then when Río-Hortega refused the invitation from Penfield to move to the Montreal Institute of Neurology^3^. The government of the Spanish 2nd Republic left the capital moving to Valencia on November 6, 1936. By November 23, the government ordered an evacuation to Valencia of all civil servants (including University professors and other intellectuals), not directly involved in the defense of Madrid: disobedience implied the risk of cease and loss of academic position (Terán and Redondo, 2007)^4^. It was the brand-new Ministry of Health, the known anarchist leader Federica Montseny, who ordered Pío del Río-Hortega to move to Valencia, too. Before leaving Madrid, Río-Hortega tried to convince Jorge Francisco Tello and Fernando de Castro to join him, but they decided to remain in Madrid, defending the Cajal Institute (Vial, 1996; de Castro, 2019). Asunción Amo, her aunt Felisa (Pío’s sister), and Nicolás Gómez del Moral arrived in Valencia in January 1937 following the researcher. However, the situation was not easy there either: Pío del Río-Hortega barely spent a couple of months at the Mediterranean city, and although he was tasked with propagandistic duties, he had some time to spend at the Faculty of Medicine to work in the lab of his friend Luis Urtubey (Professor of Pathology, there), Alcover, and Campos.

Since the end of the 1920s, Pío del Río-Hortega has been on the top of Neuropathologists, the reason for which he received offers from many different countries to move there to work and then overcome the difficult times of Spain. In January 1937, Río-Hortega moved to Paris together along with his reduced “exiled court” (Asunción Amo, his sister Felisa, and Gómez del Moral), accepting an invitation of the reputed neurosurgeon Clovis Vincent (1879–1947) to work at the Histopathology Laboratory of the Hôpital de la Pitié-Salpêtrière. Vincent, a worldwide specialist in the treatment of meningiomas and hypophisarian tumors, had provided for years the Spanish neurohistologist with numerous biopsies of different neural tumors for his classification of nervous tumors (del Río-Hortega, 1932; Ramon and Cajal Agüeras, 2016). Pío del Río-Hortega, his pupil Isaac Costero, Asunción Amo del Río, and Henry Berdet (Vincent’s assistant) worked in a very small room with just one modern microscope, trying to complete the classification of the brain tumors (del Río-Hortega Bereciartu, 1993). It is interesting that while being in Paris, Pío del Río-Hortega was nominated for the second and last time for the Nobel Prize in Physiology or Medicine 1937 by his former colleagues from the University of Valencia, Luis Urtubey and José Puche Álvarez, again for Río-Hortega’s *“work on glia tissue (microglia and oligodendroglia)”^5^*. Unfortunately, the technical conditions at that clinical space in Paris were not good for scientific research, and Río-Hortega decided soon to move.

### Río-Hortega’s Exile in Oxford: Dorothy Russell

Being in Paris, there was a second offer from Penfield, as well as from other disciples to move to Mexico, Cuba, or Venezuela, but Pío del Río-Hortega decided to accept the generous offer by another internationally reputed neurosurgeon, the Australian-born Hugh Cairns (1896–1952). After his time at Harvard with Harvey Cushing, Cairns came back to Oxford to set up the *Nuffield Department of Surgery*, and in 1937, he offered Río-Hortega to build a piece-by-piece replica of “The Transatlantic,” the colloquial name of the laboratory of *Don* Pío at the *Residencia de Estudiantes*. Cairns obtained for Río-Hortega the position of the main neuropathologist. Instrumental in this move to Oxford was the Canadian neuropathologist William C. Gibson, who, as said above, worked with Río-Hortega and de Castro in the spring of 1936, until the burst of the war (Gibson, 1994). Together with Río-Hortega, his sister, his niece Asunción, and his friend Nicolás Gómez del Moral moved from Paris to Oxford, arriving there on November 10, 1937. With the senior help of Gibson, Asunción Amo del Río acted as the official translator for her uncle, and immediately she returned to the lab to collaborate with Río-Hortega. She was officially contracted as 2nd class auxiliary of the laboratory, with a salary of 3 Sterling pounds/week (Figure 1B). Other illustrious Spanish refugees were also in Oxford at that time, like the medical doctor Josep Trueta^6^, the biochemist Severo Ochoa (who had aspired to join Río-Hortega’s lab in Madrid, and future Nobel laureate in 1959)^7^, the neurosurgeon Sixto Obrador^8^, the diplomat and historian Salvador de Madariaga^9^, and the bacteriologist Paulino Suárez. All the exiled Spaniards used to meet in the houses of the others, and Asunción was accepted as one more among them. Quite frequently, the physicist Arturo Duperier^10^ and the lawyer José Castillejo^11^ (who were in London) traveled to spend the weekends with the Oxonian group. In February 1939, Pío del Río-Hortega was named doctor *honoris causa* in Science by the University of Oxford^12^, the ceremony that was expressly attended by the Nobel laureate in Physiology or Medicine 1932, Sir Charles Sherrington. The work by Río-Hortega and Asunción Amo was completely focused on the classification of neural tumors (Figures 1C,D), and she also collaborated in the translation into English of the classification he published years before (del Río-Hortega, 1934). In recurrent reports, Cairns highlighted the quality of the work developed by Río-Hortega and Amo as superior to others. Indeed, she was offered to continue, but she rejected the contract: the bloody civil war had ended and Asunción Amo del Río and her aunt Felisa decided to come back to Spain, finally leaving to Valladolid by the end of June 1939 (del Río-Hortega Bereciartu, 1993). After 17 years of scientific involvement with his uncle, Asunción abandoned the United Kingdom, went back to Spain, got married, and never returned to the laboratory. Río-Hortega and Gómez del Moral decided to remain in Oxford.

**FIGURE 2:**
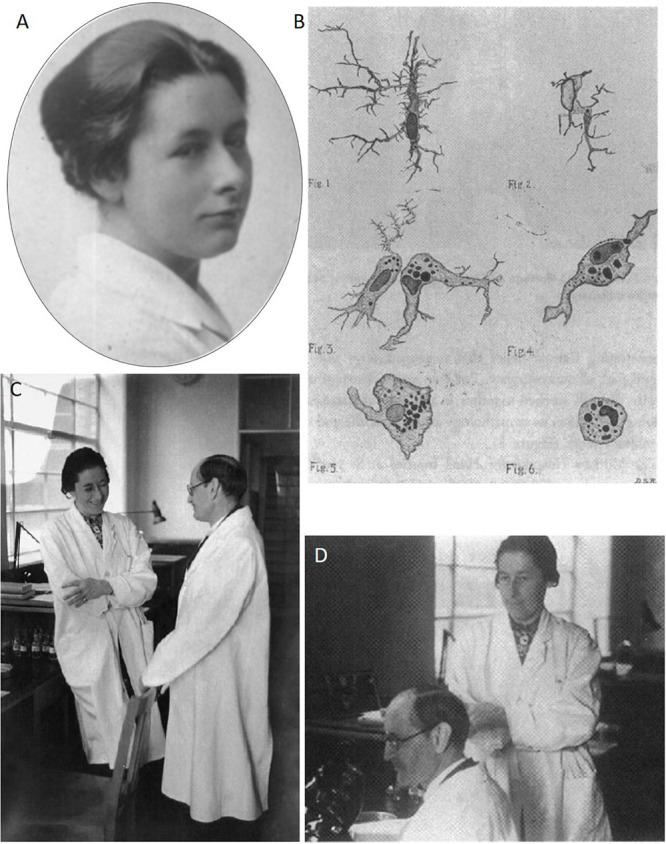
Dr. Dorothy Russell. **(A)** Young Dorothy Russell circa 1919, while studying at The London Royal Hospital (taken from Women at Queen Mary Exhibition Online). **(B)** Original drawing of microglial and macrophage cells done by Dorothy Russell at Wilder Penfield’s laboratory and published in Russell (1929). **(C,D)** Dorothy Russell and Pío del Río-Hortega at the latter’s laboratory at the University of Oxford (1939–1940).

With the burst of the 2nd World War and for the entire wartime, Hugh Cairns organized a Neurosurgical Head Injury Unit for the Army, physically emplaced at St. Hugh’s College, the unit that was joined by Pío del Río-Hortega since September 1 (Geddes, 1998). Cairns also recruited one of his main collaborators and friends, the also Australian-born (like Cairns) British pathologist Dorothy Russell (1895–1983; Scolding, 2004). Dorothy Stuart Russell was born in Sydney on June 26, 1895, as the second daughter of the bank clerk Phillip Russell and his wife, Alice Cave. After the premature death of her father in 1898 of pulmonary infection, his widow and descendants move to Queensland, where the mother remarried and had a third child. Due to economic difficulties, in 1904, Dorothy Russell and her older sister were sent to England, where their father’s sister was vicar at Fowlmere (Cambridgeshire), who took both nieces in charge. Dorothy Russell joined the *Perse School for Girls* (Cambridge) in 1908. Her interest in research dates back to her time as a schoolgirl. In the words of Barbara Adams subsequently Baroness Wootton, the distinguished sociologist and criminologist Dorothy’s companion in her student days, “*She knew just what she wanted. She was determined to be a doctor, not a practising doctor, but a research doctor*” (Geddes, 1997). Very skilled in Botany, once at the University of Cambridge, Dorothy chose the Natural Sciences Tripos and won the first degree in Zoology in 1918 (Figure 2A). It was not up to 1942 that she won her BA since it was not allowed to women (Geddes, 1997). This mixture of perseverance and obstacles saw an open window when, because of the 1st World War and the shortage of medical doctors, women were encouraged to study Medicine, and *The London Hospital Medical College* had reluctantly started accepting women in 1918. Despite the initial opposition of her uncle, Dorothy Russell left Cambridge in 1919 to be one of the 30 women accepted in *The London*. There, she was immediately interested in Pathology, one of the strengths of *The London* due to Prof. Turnbull. Once Dorothy Russell got her M.D. degree (1922), she joined Turnbull’s Institute of Pathology, where she researched for the next years, mainly in renal physiology and kidney diseases. She published some papers (Russell, 1923; Bayliss et al., 1933), and the special report “A classification of Bright’s disease,” published by the Medical Research Council in 1929, which received the gold medal of the University and was the basis of Russell’s MD thesis 1 year later (Geddes, 1997). However, not less important for her and the purpose of the present work was there she had the chance to know Hugh Cairns. At that time, he was a young neurosurgeon in love with the tight work between neurosurgeons and neuropathologists that he had seen in Boston, personalized by the Cushing (neurosurgeon) and Bailey (neuropathologist) duet.

Russell received a remarkable baptism on neurophysiological techniques during 1929 when she was a Rockefeller fellow in Montreal (Canada) to work with Wilder Penfield: he taught Russell the techniques of Río-Hortega (in some cases, partially modified by Penfield). There, she published interesting work on how to stain microglia *in vitro* with Trypan Blue (Figure 2B; Russell, 1939; Geddes, 1998). This period working at McGill University was seriously imprinted in Dorothy Russell and, before arriving in Oxford, the nervous system was already the main subject of her research, including, of course, the CNS tumors (Bayliss et al., 1933; Russell and Ellis, 1933; Russell and Tallerman, 1937; Russell, 1939). In 1935, Dorothy Russell was the first to grow glial tumor cells in culture. While working in the histopathology laboratories of St Bartholomew’s and The London with J. O. W. Bland (who sadly died before the publication of their findings), they used Ronald Canti’s time-lapse cine microscopy techniques to record pulsatile contractions of human, tumor-derived oligodendrocytes, and other living glioma cells. Russell showed the cine film to an audience of neuropathologists in New York on December 27, 1935: “Penfield was in the audience and found it a very thrilling experience” (Scolding, 2004). During these years, her former labmate at Turnbull’s laboratory, Hugh Cairns, was repeatedly treated to recruit Russell as a neuropathologist, but she refused, considering herself as a general and not a hyper-specialized pathologist.

At the 2nd World War outburst, Dorothy Russell finally joined the then British Army’s Brigadier Hugh Cairns and his Neurosurgical Head Injury Unit. Then, she became the main direct collaborator of Pío del Río-Hortega until he left Oxford for South America (see below), and even succeeding him as the head of Neuropathology at the Radcliffe infirmary. Both work hard together, and it was an outstanding opportunity for Russell to further complete her skills with the metallic impregnations devised by the Spanish Neurological School (for technical aspects of these, see de Castro and Ramon y Cajal, 1933). During the 2nd World War, and besides the clinical activity, Dorothy Russell published three scientific papers, including her first one on hydrocephalus and one regarding the effects of antibacterial compounds (Clark and Russell, 1940; Pennybacker and Russell, 1943; Russell and Beck, 1944). Besides, she published four more papers just at the end of the war (Beck and Russell, 1946; Cairns and Russell, 1946; Russell, 1946a,b). Indeed, she was also involved in the preclinical studies with penicillin (Cairns and Russell, 1946; Schiller et al., 1948) that was isolated in Oxford by Fleming, Florey, and Chain^13^. In 1944, Dorothy Russell was the first woman to achieve the status of Professor and Chair of Pathology all over Europe. Contemporarily, her former master, Prof. Turnbull, became chair of Morbid Anatomy at The Royal London, the same hospital in which she was one of the first women medical students (Scolding, 2004).

Most of Río-Hortega’s time in Oxford was devoted to work as a clinical neuropathologist and classifying the tumors of the nervous system, working together with Dorothy Russell (Figures 2C,D), although they never published together. This time of wars, anxiety, and instability resulted in a low production for Pío del Río-Hortega: (i) one scientific paper in *The Lancet* (del Río-Hortega, 1939) and (ii) one paper devoted to divulgation (it can be even considered as propagandistic) at the beginning the Spanish Civil War (del Río-Hortega, 1937). It would be necessary for him to find a new oasis of calm at the other side of the Atlantic Ocean to boost his scientific production again. That would be the last station in Río-Hortega’s exile and scientific career.

Dorothy S. Russell died years later, in 1983. In the words of Barbara Boucher, one of her students and a researcher at the Institute of Cell and Molecular Science, *Dr. Russell was a great person, special, meticulous, accurate, a brilliant speaker, and highly honest^14^*.

### Río-Hortega’s Final Exile in Argentina and Amanda Pellegrino de Iraldi

Once the bombs started bursting too close to Oxford, Pío del Río-Hortega decided to accept a proposal to give some courses and move to Buenos Aires (Argentina), where he arrived on August 28, 1940. Already in 1925, he had visited Argentina and Uruguay, invited by the *Institución Cultural Española de Buenos Aires*, to give a course on Histology: that was a success and Río-Hortega always kept it in mind. In 1940, some of his pupils (Moisés Polak) and old friends (Bernardo Houssay, future Nobel laureate in Physiology or Medicine 1947) organized Río-Hortega’s tour. As happened in Oxford, the *Institución Cultural Española de Buenos Aires* built a replica of Río-Hortega’s laboratories at Madrid, opened in 1941, and together decided to call it *Laboratorio Ramón y Cajal*. It was a great moment for Pío del Río-Hortega: he returned to the first row of scientific research, after the forced break derived from the wars in Europe. Río-Hortega started to recruit new collaborators for his brand-new laboratory and then work in the Peripheral Nervous System and neural tumors.

**FIGURE 3:**
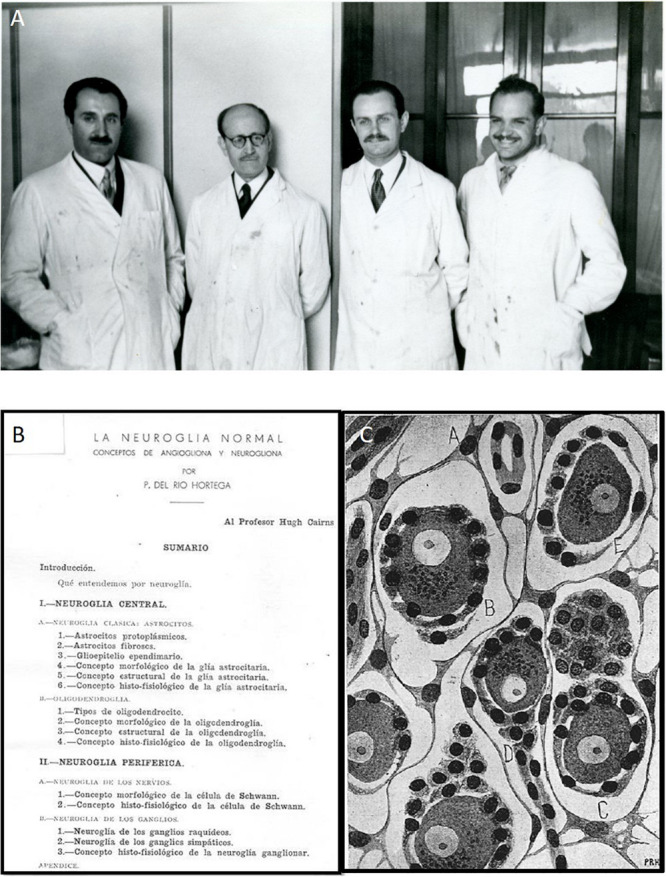
Pío del Río-Hortega in Argentina. **(A)** From the left to the right, Prof. Washington Buño, Dr. Pío del Río-Hortega, Moisés Polak, and Dr. Crosso, in Buenos Aires. **(B)** Front page of Río-Hortega’s paper entitled “The normal neuroglia. Concepts of ‘angiogliona’ and ‘neurogliona,”’ published in 1942 in honor of Hugh (del Río-Hortega, 1942). **(C)** Glial cells (*A*, *C*) and sensitive neurons (*B*, *D*, *E*) from the sensitive ganglia (del Río-Hortega et al., 1942). See on the bottom right corner one of the rare signatures of Pío del Río-Hortega in his original drawings (as: P.R.H.).

Among these new disciples in Argentina, we should highlight the names of Moisés Polak (1910–1981; Figure 3A), José Manuel Prado, Washington Buño^15^ (1909–1990; Figure 3A), Pardiñas, David Celener, León Zimman, Aranovitch, Manuel Ojea, and, chronologically the latter but the main one for our present work: Amanda Pellegrino de Iraldi (1917–2007). Born from Italian immigrants in San Nicolás (Argentina), she studied Medicine in Buenos Aires and met Pío del Río-Hortega, becoming his last direct disciple. Pellegrino learned Histology from the exiled Spaniard and her husband, Dr. Carlos Iraldi^16^, who was the GP supervising the medical problems of Río-Hortega, together with Dr. Avelino Gutiérrez. Pío del Río-Hortega had founded the journal *Archivos de Histología Normal y Patológica^17^* from the University of Buenos Aires (1942), a journal in which he published his works on the structure of the sympathetic and sensitive ganglia and, mainly, related to tumors of the CNS and PNS, coining the concepts of angioglioma and neuroglioma (Figures 3B,C). During his almost 5 years in Argentina, he achieved 22 papers and one book, some of them when he was hospitalized (del Río-Hortega, 1944, 1945a,b). However, Río-Hortega was seriously ill and finally died on June 1, 1945. Among the incredible list of top authors writing obituaries at the death of *Don* Pío, we want to highlight that of his younger labmate Fernando de Castro: *“He* [Río-Hortega] *owes the most beautiful discoveries, among them, the glial component of the nervous centres.* […] *He was one of the few world morphologists with great analytical perspicacity”* (de Castro, 1981).

The hole left by the death of Río-Hortega was substantial and urged the *Institución Cultural Española de Buenos Aires* to offer to another brilliant disciple of Cajal to substitute *Don* Pío as the head of the *Laboratorio Ramón y Cajal*: Fernando de Castro. Although the hard postwar times in Spain tempted him, Castro’s final decision was to remain in Madrid: the familial links and the responsibility with Cajal’s heritage were very important to him (Gomez-Santos, 2009).

**FIGURE 4:**
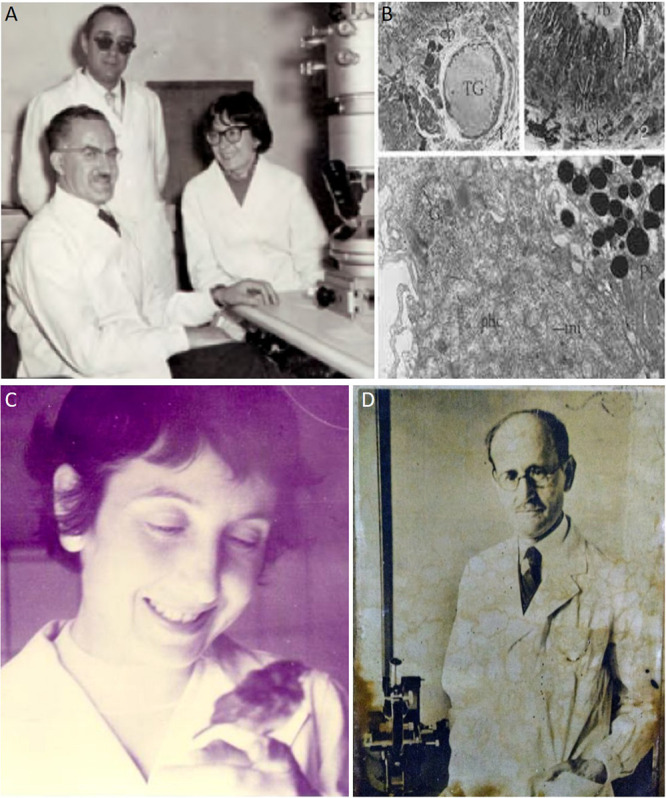
Prof. Amanda Pellegrino de Iraldi. **(A)** Amanda Pellegrino de Iraldi in 1960 at Buenos Aires, together with Prof. Eduardo De Robertis (seated on the left) and Dr. Pedro Antonio Rodríguez-Pérez (on feet), commissioned by the Instituto Cajal (Spain) for the acquisition of an electron microscope like the one in the picture. **(B)** Images of synaptic vesicles isolated from nerve terminals taken at the electron microscope from sections of rat brain, originally published by De Robertis et al. (1962) in Nature. **(C)** Young Amanda Pellegrino de Iraldi admires a small bird in her hands. **(D)** Reproduction of the original portrait of Pío del Río-Hortega that Prof. Pellegrino de Iraldi had in her bureau. See the effects of time in the photograph.

Far from finishing with the death of his master, the scientific career of Amanda Pellegrino de Iraldi had just started. As Dorothy Russell, she never published work together with Pío del Río-Hortega, but after working in the interaction of atherogenesis and the endocrine system (Hojman et al., 1959; Malinow et al., 1959, 1960)^18^, she developed an important scientific and academic career in Neuroscience as a disciple of Eduardo De Robertis (Figures 4A,B; De Robertis et al., 1965).

In a magnificent series of papers, she contributed to the identification of the adrenergic synaptic vesicles by electron microscopy (Figure 4B). Furthermore, she demonstrated the coexistence of different neurotransmitters in the same synapse (Hojman et al., 1959; De Robertis et al., 1961a,b; De Robertis, 1962; De Robertis et al., 1962, 1963, 1965; Pellegrino de Iraldi and De Robertis, 1962; De Robertis et al., 1963)^19^. The identification of the synaptic vesicles was the definitive confirmation of the individuality of neurons raised by Cajal with his Neuronal Theory. Among the first publications of Amanda Pellegrino de Iraldi were two collaborations with Antonio Pedro Rodríguez-Pérez (1912–1964; Figure 4A), one secondary character in the Spanish Neurological School who joined the Cajal group via Fernando de Castro and, at that time, was transiently working again for the *Instituto Cajal* after years of exile in Venezuela (Rodríguez Ruiz, 2013). Just when Amanda Pellegrino de Iraldi returned to Buenos Aires from her stage in Sweden with Prof. Arvid Carlsson^20^ to learn fluorimetric techniques for the measure of catecholamines, Rodríguez-Pérez arrived at Buenos Aires to evaluate the purchase of an electron microscope for the *Instituto Cajal*. From these few months of collaboration, they published two papers on the corpuscles of Grandy and Herbst, respectively, in the journal of the *Instituto Cajal* (Pellegrino de Iraldi and Rodriguez-Perez, 1961; Rodriguez-Perez and Pellegrino de Iraldi, 1961). With these, Pellegrino de Iraldi closes a circle with the School of Cajal (Figure 4D).

Amanda Pellegrino de Iraldi undoubtedly was the favorite mentee of Eduardo De Robertis (Bunge, 2016). Together with De Robertis, Pellegrino de Iraldi published a total of 24 scientific publications, mostly devoted to the electron microscopic study of different CNS structures, with particular attention to synaptic vesicles and the associated neurotubular system [see Pellegrino de Iraldi and De Robertis, 1968, 1970). At the death of Eduardo De Robertis, Dr. Pellegrino de Iraldi was his successor as Director of the *Instituto de Biología Celular* (Buenos Aires; Figures 6A,B), and she developed a successful research performance mainly focused in this line, until her last publications in 2003. She collaborated for years with her co-disciples of De Robertis, Georgina Rodríguez de Lores Arnáiz, and Luis M. Zieher (publishing seven and two papers together, respectively). Among the direct mentees of Amanda Pellegrino de Iraldi, we should highlight Ángela María Suburo (with whom she published eight papers), Guillermo Jaim Etcheberry (four papers together), Roberto Gueudet (they shared three papers), Carlos J. Tandler (four papers in collaboration), Juan Pablo Corazza (five papers together), Jorge Pecci Saavedra, Juana Pasquini (two papers together), Soto, Claudio Cuello (a prominent Argentinian neuroscientist that became Chair of the department at McGill University, Canada), or the Spanish Margarita Heredia (one paper together)^21^. Her academic career was also highly relevant, including the publication of an important textbook for medical students, “Histología Médica” (Saavedra et al., 1979). Amanda Pellegrino de Iraldi died in 2007. Collaborators and those students who were lucky enough to receive Amanda’s teachings during her long academic career remember her in the words of her pupil, Guillermo Jaim Etcheverry (Rector of the University of Buenos Aires from 2002 to 2006): “*These lines intend that the thousands of young people who had contact with Amanda during her long teaching career remember her unmistakable presence in our classrooms, her tenacity, her creativity, her dedication, her gift of people. That is the best tribute that a teacher can receive: the intimate recognition of those who, like me in this case, have had the privilege of collecting his teachings on science and life. Amanda, thank you.*”

### Río-Hortega is Back in Spain

At the beginning of the 1980s, the chair of Histology at the University of Valladolid, César Aguirre Viani (a direct disciple of Fernando de Castro), visited Argentina and established contacts to repatriate Pío del Río-Hortega’s mortal remains to Spain. This finally happened in 1986 (Figure 6C), when King Juan Carlos I had already transformed Spain into a liberal democratic system. Two people were entrusted to receive the remains of *Don* Pío at Valladolid: Río-Hortega’s former collaborator and niece Asunción del Río, and the Nobel laureate, friend, and rendered admirer of *Don* Pío, Severo Ochoa (see above for details on Severo Ochoa; Figure 6D). Río-Hortega’s mortal remains stayed at the Pantheon for Illustrious at Valladolid. In August 1995, Asunción del Amo del Río died in Manzanares (Ciudad Real), where she had lived since her marriage with the judicial secretary of this town (Figure 6E).

## Discussion

Here, we describe the female contribution to the School of Pío del Río-Hortega. The first character, Asunción del Río, was a technician with limited impact on Science. However, it is important to highlight that she was the person who collaborated with Pío del Río-Hortega during the darkest moments of his scientific career and life. This collaboration was probably essential for *Don* Pío to perseverate in research during his difficult times at Valencia, Paris, and Oxford. The figure of laboratory technicians or laborers, generally women, was very common at the time, included in the Cajal *Laboratorio de Investigaciones Biológicas*. They assisted in making the histological preparations and they were considered members of the laboratory in their own right. Not in vain, female technicians appear in many pictures of the Cajal (Giné et al., 2019). The case of Manuela Serra, a Cajal worker, stands out. She was included in the list of the directors or lead researchers of projects commissioned by the JAE at the *Laboratorio de Investigaciones Biológicas*, and she became the single author of a scientific article (Serra, 1921; Giné et al., 2019). We think that the role of Asunción del Amo in the laboratory of Pio del Rio-Hortega could be equivalent to that of Manuela Serra in that of Santiago Ramón y Cajal. Although Asunción del Río was not the author of any scientific paper published, it is important to highlight that she was the person that collaborated with Pío del Río-Hortega during the beginning of his career and during his exile in Europe. This collaboration was probably essential for *Don* Pío to perseverate in research during his difficult times at Valencia, Paris, and Oxford.

**FIGURE 5:**
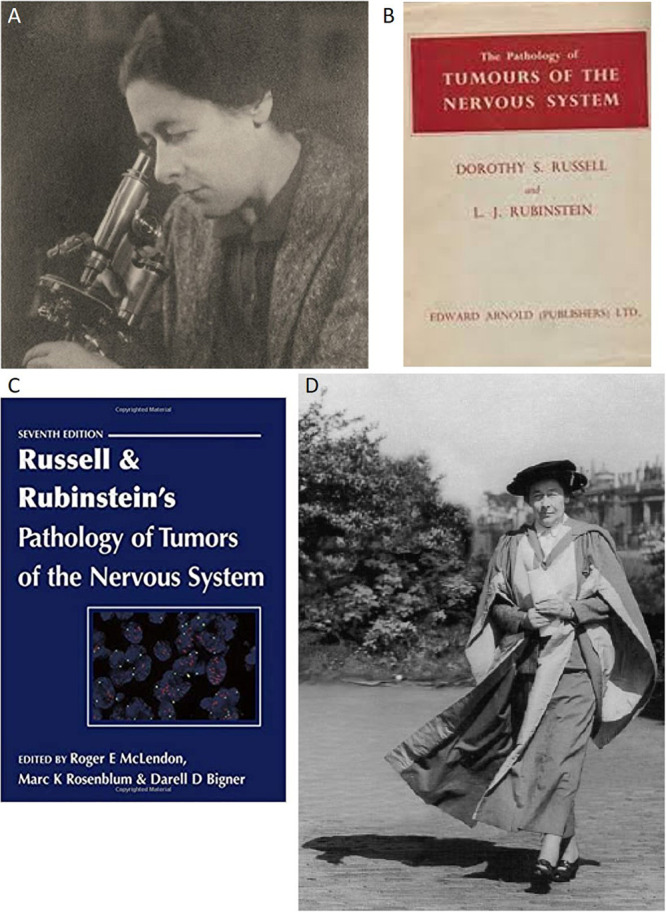
The women disciples of Río-Hortega after the death of *Don* Pío (I). **(A)** Dr. Dorothy Russell at the microscope, during her scientific maturity. **(B)** First edition of the textbook on the tumors of the brain by Russell and Rubinstein (1959). **(C)** Fifth edition of the Russell and Rubenstein textbook (Russell and Rubinstein, 1989). **(D)** Dorothy Russell receives her doctor *honoris causa* from the University of Glasgow (1951).

After the 2nd World War and back in *London*, Dorothy S. Russell made outstanding achievements as a neuropathologist (Figure 5A). Her work was focused on the tumors of the nervous system, hydrocephalus, intracranial hemorrhages, and metabolic conditions derived from tumors in the hypothalamus–hypophysis axis and pituitary gland; the latter may also have been significantly influenced by Pío del Río-Hortega, one of the pioneers in the study of the pituitary tumors. Godwin Greenfield (National Hospital at Queen Square) and Dorothy Russell, knowing the complexity of the nervous system and the special techniques for its study, forced the specialization and, consequently, the need for clinical specialists in Neuropathology, which expanded all over the United Kingdom with the advent of the National Health Service in 1948 (Allen, 2006). After the death of Río-Hortega, Dorothy Russell was Sir Hugh Cairns’ favorite neuropathologist and became a worldwide leader in the field of CNS tumors, an achievement in which the use of the silver methods from the Spanish Neurological School was very important, and with the collaboration of one of her younger disciples, Lucien Rubinstein, she published the first textbook focused on Neurooncology Russell and Rubinstein (1959). This influential treaty was repeatedly published, and the World Health Organization (WHO) based its working classification of neuronal/neuroblastic tumors of the CNS on the fifth edition of (Russell and Rubinstein, 1989; Figures 5B,C). The Oxonian influence of Pío del Río-Hortega on Dorothy Russell’s views on the tumors of the CNS seems quite evident from the perspective of current Neuropathology, and the debate around the nature of medulloblastoma or cerebellar neuroblastoma is at the core of this influence (Katsetos et al., 2003). In this dispute, the use of Cajalian metallic impregnations by Río-Hortega and two of his most important disciples, Wilder Penfield (in North America) and Moisés Polak (in South America), was a determinant. The knowledge and use of these techniques (relatively delicate protocols that give rise to sometimes capricious results, in terms of frequent staining of a part of the cells but not all of them, which would represent a limitation but, at the same time, allow better identification of details than the staining of thousands of cells per field) were decisive for Dorothy Russell in the face of the widespread use of aniline dyes, more constant but also with more limited results (del Río-Hortega, 1932, 1945a; Polak, 1967; Katsetos et al., 2003). Regarding the Río-Hortega/Penfield/Russell triangle, perhaps there are no better words than those from Neil Scolding: “*What extraordinary clinical scientists they were, and how their careers coincided! All were moulded by the odd combination of the great European wars and Oxford. All shaped the future of both glial biology and their clinical fields*” (Scolding, 2004). The influence of the metallic impregnations and the concepts raised by Río-Hortega was so relevant to Russell, who published a paper on “microgliomatosis” (Russell et al., 1948): different authors, including Polak, had first proposed the existence of this type of tumor that was not accepted by Pío del Río-Hortega. Russell’s observations maybe were highly malignant gliomas with a very important necrotic content and therefore abundant microglia, or even not properly characterized lymphomas: in any case, the current international consensus is that, as Río-Hortega maintained along with his life, there are no neoplastic tumors derived from microglial cells. Besides the prizes cited above, Dorothy Russell was named doctor *honoris causa* by the University of Glasgow (Figure 5D) and by the McGill University, and the Royal College of Physicians granted her the Oliver-Sharpey Prize in 1968.

**FIGURE 6:**
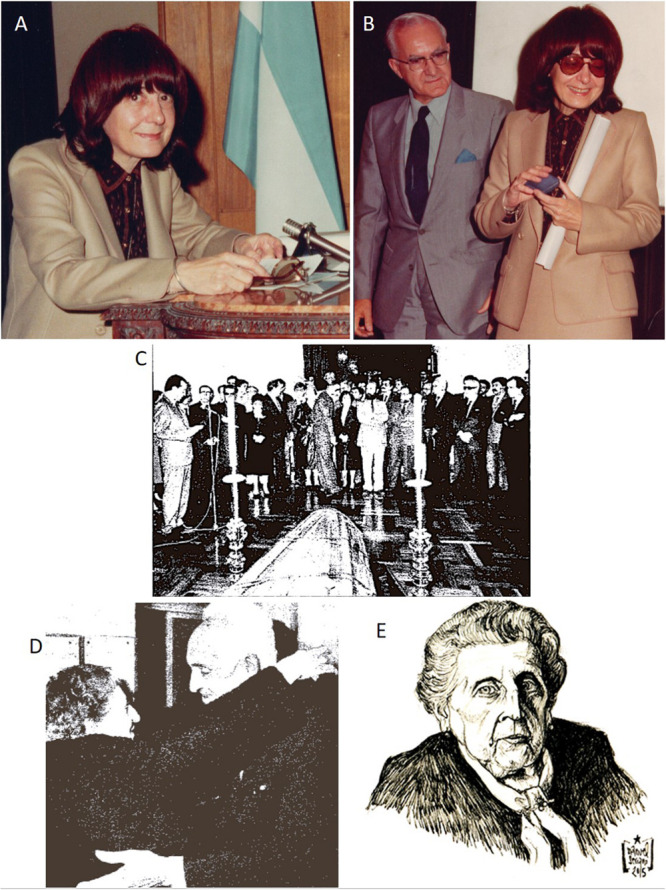
The women disciples of Río-Hortega after the death of *Don* Pío (II). **(A,B)** Prof. Amanda Pellegrino de Iraldi substitutes Prof. Eduardo De Robertis (with her in **B**) as Director of the Instituto de Biología Celular of the Universidad de Buenos Aires. **(C)** Reception of the mortal remains of Pío del Río-Hortega at Valladolid city town-hall (1986). Source: *Archives at the newspaper ABC*, issue published on October 14, 1986. **(D)** Asunción Amo del Río (on the left) embraces her old friend Prof. Severo Ochoa (Nobel laureate 1959) during the reception of the mortal remains of Pío del Río-Hortega in Valladolid (1986). Source: *Archives at the newspaper ABC*, issue published on October 14, 1986. **(E)** Portrait of Asunción Amo del Río in her last days in Manzanares (Ciudad Real) in 2015.

The third character of our work, the Argentinian Amanda Pellegrino de Iraldi, displayed a remarkable scientific career from the 1960s to 2003 (date of her last scientific publication). Before becoming herself an independent researcher with important scientific production, she closely collaborated with Eduardo De Robertis (one of the fathers of electron microscopy in the CNS) and with the Nobel laureate (2000) Arvid Carlsson (one of the pioneers working with dopamine and its influence in Parkinson’s disease). For these achievements, her initial training with Pío del Río-Hortega was so critical to learning directly Río-Hortega’s method and other important techniques for Neurohistology. We should emphasize that Amanda Pellegrino de Iraldi was the last direct pupil formed by Pío del Río-Hortega and perhaps Eduardo De Robertis’ favorite, up to the point to be nominated as the successor of the latter as Director of the Instituto de Biología Celular (Buenos Aires; Figures 6A,B). As we highlighted in the case of Dorothy Russell, along with the research performance of Amanda Pellegrino de Iraldi, we recurrently find different Horteguian subjects: glial cells, glial tumors, and hypophysis. After the fructiferous scientific offspring of his main disciple, Wilder Penfield (in the Montreal Institute for Neurology and other research institutions in Canada and the United States), Río-Hortega’s seeds were also indirectly planted in North America via his later disciple, Amanda Pellegrino de Iraldi: her most notable disciple, the also Argentinian pharmacologist Claudio Cuello, established at McGill University (Montreal Canada) from where he developed an outstanding research performance (Cuello, 2019).

There are differences and common characteristics between the three protagonists of the present work and other researchers who work during the same period and in the same geographical spaces. As for the case of Asunción Amo del Río, it is very comparable to that of Manuela Serra (Giné et al., 2019; Nombela et al., 2020): both women were born in Spain and worked as specialized technicians with Cajal (Serra) and Río-Hortega (Master of the River), with whom he recognized the talent of these women. Although Asunción Amo del Río had the relevant position of training visitors to his uncle’s laboratory, he never published a research publication, while Manuela Serra did (1921), although his work with Cajal was shorter than that of Amo with Río-Hortega. In both cases, they withdrew from the investigation when they married to start a family.

Dorothy Russell has the same Australian origins as the first woman who worked with Cajal, Laura Forster, as well as a very remarkable performance. As previously stated, Russell became a renowned neuropathologist and the first woman to hold a professorship at the British University and in Pathology, globally. For this, she has been included among the 10 most influential pathologists of all time, along with Americans Myrtelle May Canavan (1879–1953, who described the disease with her last name), Sophie Spitz (1910–1956), and Elizabeth Stern (1915–1980); Canadians Maud Menten (1879–1960, whose name is associated with the Michaelis–Menten equation for enzyme kinetics) and Georgina Hogg (1916–2002); the Hungarian-born cytopathologist at Mass General Hospital Priscilla Dienes Taft (1917–2002); the Polish Maria Magdalena Dabska (1921–2014); and the still-living Elaine Jaffe and Sharon Weiss^22^.

The case of Amanda Pellegrino de Iraldi is not an isolated case in South America and, more specifically, in Argentina, where other women passionately dedicated themselves to scientific research. This was the case of Eugenia Sacerdote de Lustig (1910–2011), born in Turin, a disciple of Giuseppe Levi like his cousin Rita Levi-Montalcini (future Nobel Prize in Physiology or Medicine), who fled the fascist regime due to her condition of a Jewish family. Contemporaries, both the Sacerdote de Lustig and Pellegrino de Iraldi shared their passion for histology and the study of tumors and worked at the University of Buenos Aires. Indeed, Eugenia Sacerdote de Lustig was a leading researcher in experimental oncology at the Ángel Roffo Institute and the Malbrán Institute (as head of Virology). Furthermore, she was a member of CONICET, published more than 180 scientific publications, and received the Hippocrates Prize (the highest distinction to be achieved by a doctor in Argentina) and the Bicentennial Medal, a distinction granted by the Senate of the Argentine Republic to the most outstanding personalities (Sacerdote de Lustig, 2005).

In our previous work, we discussed the relevance of the women neuroscientists working with Cajal and his disciples in Spain and compared them with other very relevant women neuroscientists of those times (Giné et al., 2019). All these names would serve to compare the performances of the figures at the present work, as also happens with others such as Christine Ladd-Franklin (1847–1930, United States), Margaret Floy Washburn (1871–1939, United States), Marcelle Lapicque (1873–1960, France), Nathalie Zand (1884–1942, Poland), Gabrielle Charlotte Lévy (1886–1934, France), Mary Broadfoot Walker (1888–1974, United Kingdom), Elisabeth Crosby (1888–1983, United States), Una Lucy Fielding (1888–1969, Australia), Lucja Frey (1889–1942, Poland), Alexandra Adler (1901–2001, Austria), Margaret Kennard (1899–1972, United States), Elizabeth Roboz Einstein (1904–1995, Hungary), Denise Albe-Fessard (1916–2003, France), or Marianne Fillenz (1924–2012, Romanian-born New Zealander), among other pioneering women with notable contributions to many different aspects of Neuroscience (Finger, 2002).

It is then remarkable the importance of these three women and the link with Dr. Pío del Río-Hortega, the Neurosciences (Neuropathology, in particular), and Cajal’s School. Being so, they deserved to be recognized for their work and for resembling a connection between the origin of modern neuroscience and Science today.

## Data Availability Statement

The original contributions presented in the study are included in the article/supplementary material, further inquiries can be directed to the corresponding author/s.

## Author Contributions

CN: writing, references, and submission. EF-E: critical review. EG: writing. YW: critical review. JdR-H: data mining, historical images. FdC: original idea, data mining, and writing. All authors contributed to the article and approved the submitted version.

## Conflict of Interest

The authors declare that the research was conducted in the absence of any commercial or financial relationships that could be construed as a potential conflict of interest.

## Footnotes

1 https://www.nobelprize.org/nomination/archive/show.php?id=12754. In 1929, the Nobel Committee received a particularly high number of nominations (121 nominations).

2 Pío del Río-Hortega was appointed as Head of the Research Section at the *Instituto Nacional del Cáncer* in 1928, and as Director of this institution in 1931. He was the director when the Spanish Civil War burst.

3 Attending this first offer in September 1936, Río-Hortega decided to send his disciple Isaac Costero (1903–1980) to Montreal.

4 New rules for the evacuation of professors toward Valencia were ordered along 1937 in parallel with the purge of those who did not compromise with the Republic, who were destitute from their posts as civil servants (Fernández Terán, 2014).

5 https://www.nobelprize.org/nomination/archive/show_people.php?id=2337. In 1937, the Nobel Committee received 180 nominations for this Prize. It is very interesting that despite the Spanish Civil War, colleagues proposed Pío del Río-Hortega for the Nobel Prize in 1937. Unfortunately, nobody proposed another brilliant direct disciple of Cajal, Fernando de Castro. The Belgian physio-pharmacologist Corneille Heymans (1892–1968) was awarded the Nobel Prize in 1938, and there is a general and growing consensus that this prize should be shared with Fernando de Castro. Although no one nominated Dr. de Castro for the Nobel Prize that year, the Nobel Committee considered his contribution and discussed whether he was still alive or not, since the Prize is not awarded posthumously. The relevance of the discoveries by Fernando de Castro stated both in the presentation of the Nobel Prize to Heymans by Prof. Liljestrand (Ghent; January 16th, 1940) and in Heyman’s Nobel lecture (Stockholm; December 12th, 1945; for a detailed description of this peripeteia) (Liljestrand and Koller, 1965; Gallego, 1967; de Castro, 2009a,b).

6 Josep Trueta Raspall (1897 Barcelona, Spain-1977 Barcelona, Spain) is an extremely important personality: exiled after the civil war; he was specialized in fractures and war wounds. He also joined Howard Florey and Ernst Chain while developing Penicillin and Trueta’s was the very first animal surviving after the injection with the antibiotic. He was Professor of Orthopaedics at Nuffield (1949–1966) when he retired in Spain.

7 A former collaborator of Pío del Río-Hortega at the Instituto Nacional del Cáncer in Madrid, Severo Ochoa (1905 Luarca, Spain–1993 Madrid, Spain) left Spain at the very beginning of the Spanish Civil War in search for a place safe enough to work. In this search, Ochoa and his wife worked later in Germany, United Kingdom (Oxford), and, finally, the United States. Ochoa counted with highly relevant mentors, as Otto Meyerhoff (Nobel Prize in Physiology or Medicine 1932), Otto Warburg (Nobel Prize in Physiology or Medicine 1931), Henry Dale (Nobel Prize in Physiology or Medicine 1936), and Fritz Lipmann (Nobel Prize in Physiology or Medicine 1953). In 1949, Ochoa moved to the Washington University, St. Louis, and, to the New York University in 1945 to become chair of the Biochemistry Department. He shared the Nobel Prize in Physiology or Medicine 1959 with Arthur Kornberg “*for their discovery of the mechanisms in the biological synthesis of ribonucleic acid and deoxyribonucleic acid*” (https://www.nobelprize.org/prizes/medicine/1959/summary/). He is considered one of the fathers of Molecular Biology and the genetic code. A fervent admirer of Santiago Ramón y Cajal, besides a collaborator and a friend, Ochoa was also an admirer of Pío del Río-Hortega (Gómez-Santos, 1993; Ochoa, 1999). Not in vain, the Spanish Research Council (Consejo Superior de Investigaciones Científicas-CSIC) entrusted Severo Ochoa to write the prolog for the publication of Río-Hortega’s “El Maestro y yo,” about his relationship and experience of del Río-Hortega with Cajal. The manuscript was found when the mortal remains of Pío del Río-Hortega were returned to Spain (see below) and finally published (del Río-Hortega and Insúa, 1986).

8 Sixto Obrador Alcalde (1911 Santander, Spain–1978 Madrid, Spain) arrived at Oxford in 1936, to work with Charles Sherrington. Before, in Spain, it was Pío del Río-Hortega who suggested him to become a neurosurgeon. A close friend of Severo Ochoa, both left Oxford when the first bombs of the 2nd World War forced to stop research.

9 Salvador de Madariaga Rojo (1886 La Coruña, Spain–1976 Locarno, Switzerland). He was a mines engineer by the ENS des Mines (Paris, France) in 1921 and hired by the Society of Nations. Professor of Spanish language at the Oxford University (1928–1931). He was the first ambassador of the 2nd Spanish Republic in Washington (1931) and then ambassador at the Society of Nations (1932) and in France (1932–1934). He was nominated for the Nobel Prize of Peace (1939 and 1952) and that of Literature (1952).

10 Arturo Duperier Vallesa (1896 Pedro Bernardo, Spain–1959 Madrid, Spain) became a leader in the Physics of cosmic radiation.

11 José Castillejo Duarte (1877 C. Real, Spain–1945, London, UK) was a lawyer and pedagogist, professor at the innovative Institución Libre de Enseñanza (Spain). Castroviejo was a capital figure for and in the Junta para la Ampliación de Estudios-JAE (Spain), an institution founded after Cajal’s advice at the beginning of the twentieth century and an indispensable vehicle for the formation of the Cajal School and the general progress of Science, Culture and Art in Spain up to our Civil War.

12 Río-Hortega was the second Spanish scientist receiving this distinction: the first one was Santiago Ramón y Cajal, in 1894. He was also designed honorary member of the Trinity College and lecturer of the University.

13 The first human clinical test with penicillin was carried out in the African front to determine the optimal dosage to be used in humans. Hugh Cairns and Howard Florey supervised these tests: both were commissioned to Cairo (Egypt), in 1943 (Stone et al., 2016). Hugh Cairns and Dorothy Russell published just these two papers together (Cairns and Russell, 1946; Schiller et al., 1948).

14 Interview with Barbara Boucher on the Queen Mary University of London women’s website: http://www.women.qmul.ac.uk/virtual/women/atoz/russell.htm).

15 The case of Washington Héctor Buño Vázquez is very relevant: he was originally a disciple of Clemente Estable (1894–1976), maybe the introducer of Neuroscience in South America after his years working with Santiago Ramón y Cajal, in Madrid. Already professor in Montevideo, Buño moved in 1944 to Buenos Aires to work with Pío del Río-Hortega.

16 Forty years later, the couple published a scientific paper together (Iraldi and Pellegrino de Iraldi, 1987).

17 With time, this journal became Anales de la Fundación Roux-Ocefa.

18 The very first publication by Pellegrino de Iraldi was in an Argentinian medical journal about teaching rather than research (González Bosch et al., 1948).

19 The discovery of the synaptic vesicles of neurons is one of the *must*s in the History of Neuroscience: many relevant voices are claiming that Eduardo De Robertis deserved a Nobel Prize in Physiology or Medicine just for this discovery (Bunge, 2016).

20 Arvid Carlsson shared the Nobel Prize in Physiology or Medicine 2000 with Paul Greengard and Eric R. Kandel *”for their discoveries concerning signal transduction in the nervous system”* (https://www.nobelprize.org/prizes/medicine/2000/summary/).

21 For more details, check Amanda Pellegrino de Iraldi’s Google Scholar profile (https://scholar.google.com/citations?user=z6zw1VkAAAAJ&hl=en).

22 https://pathcore.com/resources/international-womens-day-2020
